# The multi-anion environment of Bi_13_S_18_I_2_ governs the mechanism of sodium storage

**DOI:** 10.1039/d6ta06219a

**Published:** 2026-09-21

**Authors:** Xuerui Yi, Yuanzhu Zhao, Zhenyu Guo, Megha Goyal, A. Robert Armstrong, John T. S. Irvine, Maria-Magdalena Titirici, Caroline Kirk, Neil Robertson

**Affiliations:** a School of Chemistry, EaStCHEM, University of Edinburgh King's Buildings, David Brewster Road Edinburgh Scotland EH9 3FJ UK Neil.Robertson@ed.ac.uk Caroline.Kirk@ed.ac.uk; b Department of Chemical Engineering, Imperial College London South Kensington Campus, Exhibition Rd London SW7 2AZ UK m.titirici@imperial.ac.uk; c School of Chemistry, University of St Andrews St Andrews Fife KY16 9ST UK; d Advanced Institute for Materials Research (WPI-AIMR), Tohoku University Sendai Japan

## Abstract

Sodium-ion batteries (SIBs) are promising for large-scale energy storage. Alloy-type anodes such as Bi metal offer high capacity yet suffer from severe volume expansion and unstable cycling due to alloying reactions. Herein, we report a mixed-anion Bi_13_S_18_I_2_ nanorod anode synthesised *via* a mild solvothermal method, offering a unique platform to regulate the sodiation pathway. Compared with conventional Bi metal, Bi_13_S_18_I_2_ undergoes an initial conversion reaction that may convert the electrode into Bi embedded within conversion-derived Na_2_S composites. This conversion induced matrix modulates the subsequent alloying behaviour. *Ex situ* XRD analysis, together with the plateau capacity distribution, suggests that the sodiation process differs from the stepwise alloying behaviour of Bi metal. This different electrochemcial behaviour may be associated with the mixed-anion framework in Bi_13_S_18_I_2_, which results in heterogeneous local coordination environments for Bi and gives rise to a unique conversion-alloying pathway. This work highlights the important role of mixed-anion induced local environments in adjusting sodiation pathways, offering insights into the design of alloy-type anodes for SIBs.

## Abstract 2

The multi-anion environment of Bi_13_S_18_I_2_ modulates the sodium-storage pathway.

## Graphical abstract

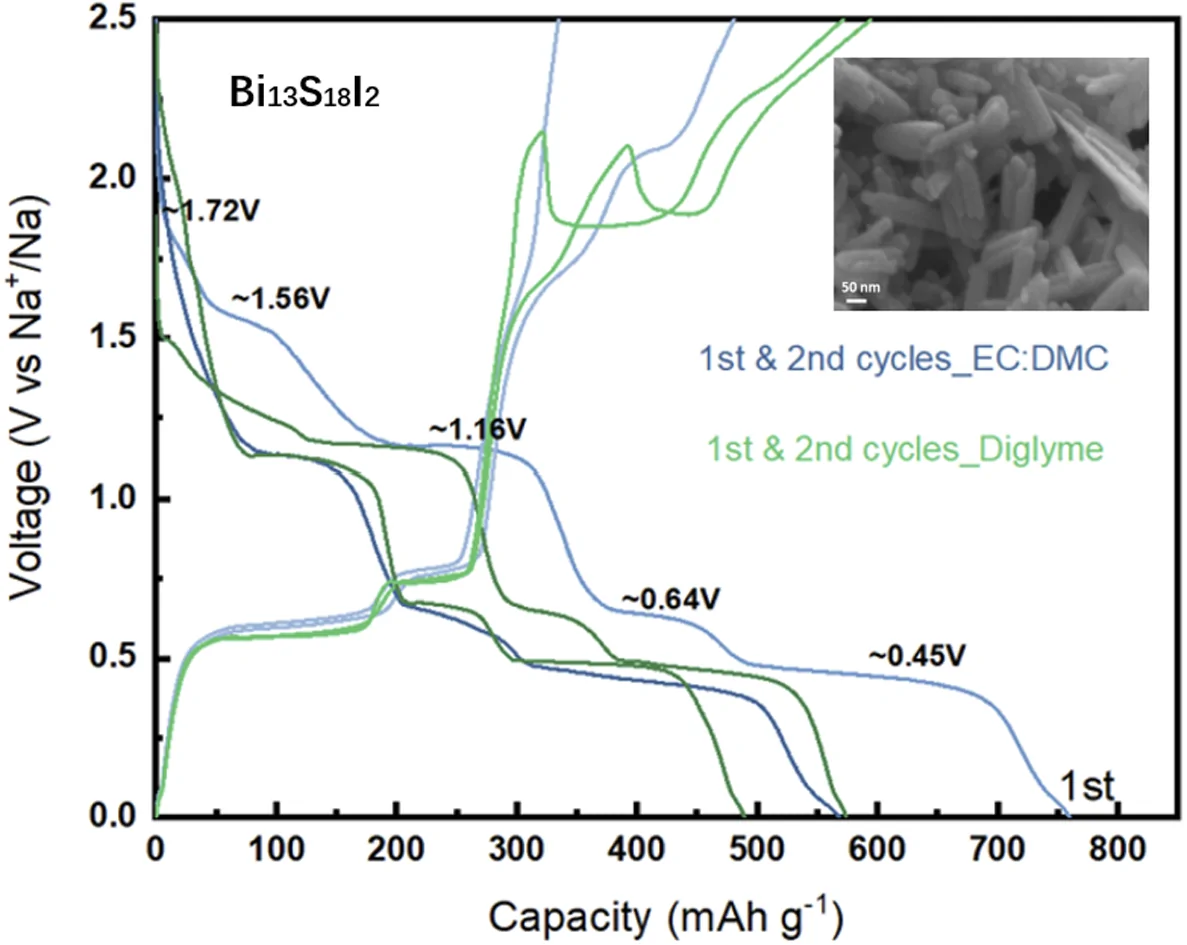

## Introduction

With the rapid growth of renewable energy integration and large-scale grid energy storage, the development of energy storage technologies with a favourable balance between cost and performance has become increasingly important.^1^ Sodium-ion batteries (SIBs) are regarded as a promising avenue because of their abundant resource, low cost, and the compatibility of their manufacturing processes with existing lithium-ion battery (LIBs) infrastructure.^2^ However, the larger ionic radius of Na^+^ often leads to sluggish diffusion kinetics and structural strain in many host electrode materials, as well as the interfacial instability during repeated insertion–extraction, which hinders the development of high-performance and long-life anode materials.^3^

Currently, anode materials for SIBs can be broadly classified into carbon-based materials, alloying-type and conversion-type materials. Hard carbon is the most widely used anode material for SIBs, owing to its good cycle stability and relatively low operating potential.^4^ Recent studies have demonstrated that rational regulation of closed-pore structures and pore architectures can further improve Na-storage behaviour, ion transport, and cycling performance in hard carbon.^5,6^ Alloying-type anodes, such as Sn, Sb and Bi, provide high theoretical capacities *via* Na-metal alloying reactions, but suffer from severe volume changes, resulting in structural pulverisation and rapid capacity decay.^7^ Conversion-type sulfide and oxide anodes can also deliver high energy density.^8,9^ However, their rigid lattice frameworks make it difficult to accommodate the strain induced by Na^+^ insertion, leading to irreversible structural collapse during the initial sodiation process. For example, Lu *et al.* reported that while Bi_2_S_3_ undergoes conversion and subsequent alloying reactions with Na^+^, its structure is not fully reversible and is rapidly degraded upon cycling resulting in fast capacity fading.^10^ In addition, Tratnik *et al.* incorporated small amounts of Bi_2_S_3_ (∼5–10 wt%) into hard carbon to enhance anode performance and further investigated the sodiation/desodiation process of Bi_2_S_3_ by *in situ* X-ray diffraction and *ex situ* solid state ^23^Na MAS NMR spectroscopy to gain insight into its reaction behaviour.^11^ However, these studies were carried out in composite electrodes with hard carbon, in which the electrochemical behaviour of Bi_2_S_3_ is coupled with the intercalation and pore filling process of hard carbon, and the overall capacity remains predominantly determined by the hard carbon component. Thus, the reaction pathways and structural evolution cannot be interpreted through the intrinsic behaviour of a single active phase.

For these reasons, investigating the conversion-alloying reaction pathways and their associated structural change in single phase systems is important for rational electrode materials design and for developing a fundamental understanding of structure–reaction relationships.^12^ Thus, it is necessary to build and study single-phase materials in which conversion-type and alloying-type reactions are embedded within the same crystal framework, so that reaction pathway selection and crystal structure change can be detected without introducing additional active phases. Compared with single-anion sulfides, such as Bi_2_S_3_, a mixed-anion system modifies the local bonding environments and charge distribution by incorporating multiple anion types, which may help to accommodate the large structural strain induced by conversion-alloying reactions and mitigate irreversible structural degradation.^13^

On this basis, mixed-anion bismuth sulfohalides of the type Bi–S–X (X = Cl, Br, I) have recently attracted increasing attention. Previous studies have reported that mixed-anion compounds, such as BiSI and BiSBr can be used as anode materials in LIBs and exhibit electrochemical features consistent with conversion and alloying reactions.^14,15^ However, these insights are typically focused on lithium-based system, and their applicability to Na^+^ systems, which involve the larger ionic radius of Na^+^ and more severe structural strain, has yet to be studied. In addition, we have previously investigated the electrochemical behaviour of mixed-anion Bi–S–I compounds in aqueous hybrid-supercapacitor systems.^16,17^ By directly tracking the changes in the Bi oxidation state using *in situ* XANES and EXAFS, we showed that Bi^3+^ can be reduced to metallic Bi^0^ under reducing conditions and partially reoxidised during the reverse scan, giving rise to pronounced redox activity. However, these studies also revealed that a fraction of Bi^0^ cannot be fully reoxidised and gradually aggregates during cycling, leading to irreversible structural changes. These results indicate that mixed-anion bismuth compounds display active redox chemistry, but whether they undergo conversion and alloying reactions under deeper reduction conditions (in secondary battery systems), and how their structures and interfaces change, remain open questions.

In this work, a simple, mild and environmentally-friendly solvothermal method was developed to prepare mixed-anion Bi_13_S_18_I_2_ nanorods without the need for high-pressure hydrothermal conditions. Compared with previously reported hydrothermal routes,^15^ this approach features shorter reaction times, milder conditions, and improved controllability. To the best of our knowledge, this is the first report of mixed anion bismuth sulfohalides as anode materials for SIBs. We further show that the Bi_13_S_18_I_2_ as anode material in SIBs undergoes a multistep sodium-storage mechanism. In the initial cycles, multiple voltage plateaus are observed, indicating the presence of complex conversion processes. From the 5th cycle onwards, two plateaus dominate, associated with Bi → NaBi → Na_3_Bi alloying reactions.^18,19^ However, the sodium storage behaviour does not strictly follow the ideal stepwise alloying mechanism observed in metallic Bi. Although similar voltage plateaus can be identified, their capacity distribution differs from the expected 1 : 2 ratio, indicating a more complex process. Moreover, for the intermediate cycles to 200 cycles, the first plateau (Bi^0^ → Bi^1−^) appears to be less pronounced while the second (Bi^1−^ → Bi^3−^) becomes more pronounced, suggesting a possible morphology change of the electrode. Upon prolonged cycling, the capacity ratio becomes closer to that of metallic Bi, accompanied by more symmetric sodiation and desodiation profiles, revealing a transition towards a simpler and more stable reaction pathway.

## Results and discussion

Fig. 1 presents the structure, morphology and compositional homogeneity of the as-prepared Bi_13_S_18_I_2_. The powder X-ray diffraction (PXRD) pattern in Fig. 1a matches well with the reference data for Bi_13_S_18_I_2_. The observed PXRD data were refined using a Pawley fitting routine. The unit cell parameters were refined in space group *P*6_3_ (*a* = 15.612 Å, *c* = 4.0168 Å, ICSD collection number 243730 (ref. 20)) with refined values of *a* = 15.643(1) Å, *c* = 4.0196 (3) Å, which is in good agreement with the published values. The observed, calculated and difference profiles from refinement of these data are presented in Fig. S1. As shown in Fig. 1c (adapted from ref. 20), the Bi_13_S_18_I_2_ is built from Bi–S ribbon-like subunits that assemble into an open, channel-containing mixed-anion framework, in which iodide ions are located at the inter-ribbon channel sites. This crystal structure of Bi_13_S_18_I_2_ results in anisotropy and distinguishes Bi_13_S_18_I_2_ from more densely packed bismuth sulfide or iodide phases.

**Fig. 1:**
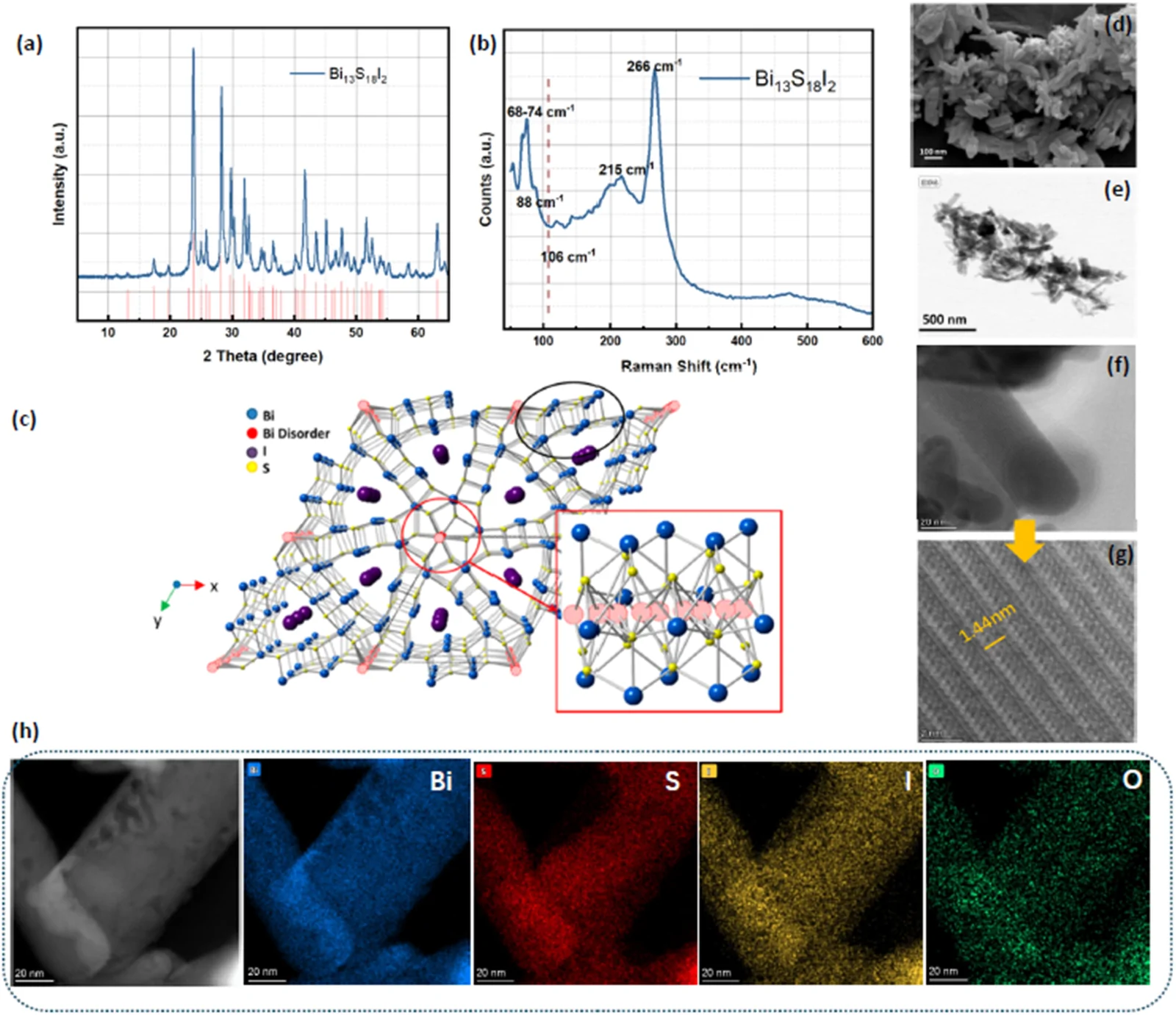
Structural characterisation of Bi_13_S_18_I_2_ sample. (a) X-ray powder diffraction pattern; (b) Raman spectrum; (c) crystal structure; (d) SEM image; (e–g) high-resolution TEM images; (h) EDS mapping.

Raman spectroscopy provided further insight into the structural framework of Bi_13_S_18_I_2_, as shown in Fig. 1b. The Raman spectrum features observed within 60–300 cm^−1^ range are typical of Bi-based chalcogenide and halide systems.^16^ A weak and broadened Raman response is observed in the 110–180 cm^−1^ region, where vibrational modes related to Bi–I (BiI_3_) and Bi–S (Bi_2_S_3_) have been reported in related systems.^21,22^ In addition, a band at approximately 266 cm^−1^ can be regarded as a fingerprint of vibrations associated with the Bi–S backbone.^23^ A weak feature appearing at around 106 cm^−1^ lies in the frequency range previously reported for Bi–Bi related vibrational modes in Bi-based chalcogenide compounds, and may be associated with Bi–Bi bonding motifs in the Bi_13_S_18_I_2_ structure.^20^ To avoid over-assignment of individual peaks, the Raman peak positions observed in this work were compared with those reported for Bi_13_S_18_I_2_, BiI_3_, and Bi_2_S_3_, as summarised in Table S1. All major Raman features fall within the reported frequency ranges of related Bi-based systems.

Electron microscopy analyses reveal a rod-like morphology of Bi_13_S_18_I_2_, with the nanorods randomly stacked into a loosely packed three-dimensional architecture (Fig. 1d and e). High-magnification SEM images in Fig. S2 display smooth rod surfaces and well-defined terminations. High-resolution TEM (HRTEM) images (Fig. 1f and g) exhibit clear and continuous periodic fringe contrasts with a spacing of approximately 1.44 nm. This enlarged periodic fringe contrast may be related to the local stacking and overlap of neighbouring nanorods observed in Fig. 1e.

The elemental composition of as-prepared Bi_13_S_18_I_2_ nanorods were investigated by SEM-energy dispersive X-ray spectroscopy (EDS) and TEM-EDS analyses. The corresponding SEM-EDS spectra and quantitative results are presented in Fig. S4 and Table S2, while the TEM-EDS spectra and compositional analyse are shown in Fig. S4 and Table S3. Both analyses collected from multiple regions consistently confirm the presence of Bi, S, and I, supporting the formation of Bi–S–I mixed anion compound. The quantified atomic percentages are not fully consistent with the nominal stoichiometry due to the inherent limitations of EDS quantification and differences in the interaction volumes of SEM and TEM. STEM-EDS elemental mapping in Fig. 1h shows a homogeneous distribution of Bi, S and I throughout the nanorods. The result indicates that I species are not present as isolated phases but are uniformly incorporated into the Bi–S framework. The oxygen signal detected likely originates from surface oxidation or adsorbed species formed during sample transfer and exposure to air, rather than from the intrinsic composition of the material. Elemental mapping, collected from two additional regions, show consistent distributions and are provided in Fig. S4. Furthermore, this uniform anion distribution is expected to lead to more uniform electrochemical reactions within particles, which can help alleviate local stress caused by volume change during Na^+^ insertion and the subsequent conversion-alloying processes.

Fig. 2 presents the porosity characteristics and surface chemical states of Bi_13_S_18_I_2_. The nitrogen adsorption–desorption isotherm (Fig. 2a) shows an uptake at high relative pressure (*P*/*P*_0_ → 1), indicating the presence of mesopores and interparticle voids arising from the packing of nanorods. The BET specific surface area is calculated to be approximately 7.98 m^2^ g^−1^, with a BJH pore volume of 0.046 cm^3^ g^−1^, suggesting a relatively compact structure of nanorod assemblies. The pore size distributions were estimated using the Barrett–Joyner–Halenda (BJH) and Dollimore–Heal (D–H) methods in Fig. 2b and c. Both analyses show that the major pore volume contribution occurs at larger pore radii, which is likely associated with interparticle voids arising from nanorod packing. The zoomed-in profile in Fig. 2c further indicates minor contributions in the small-mesopore region.

**Fig. 2:**
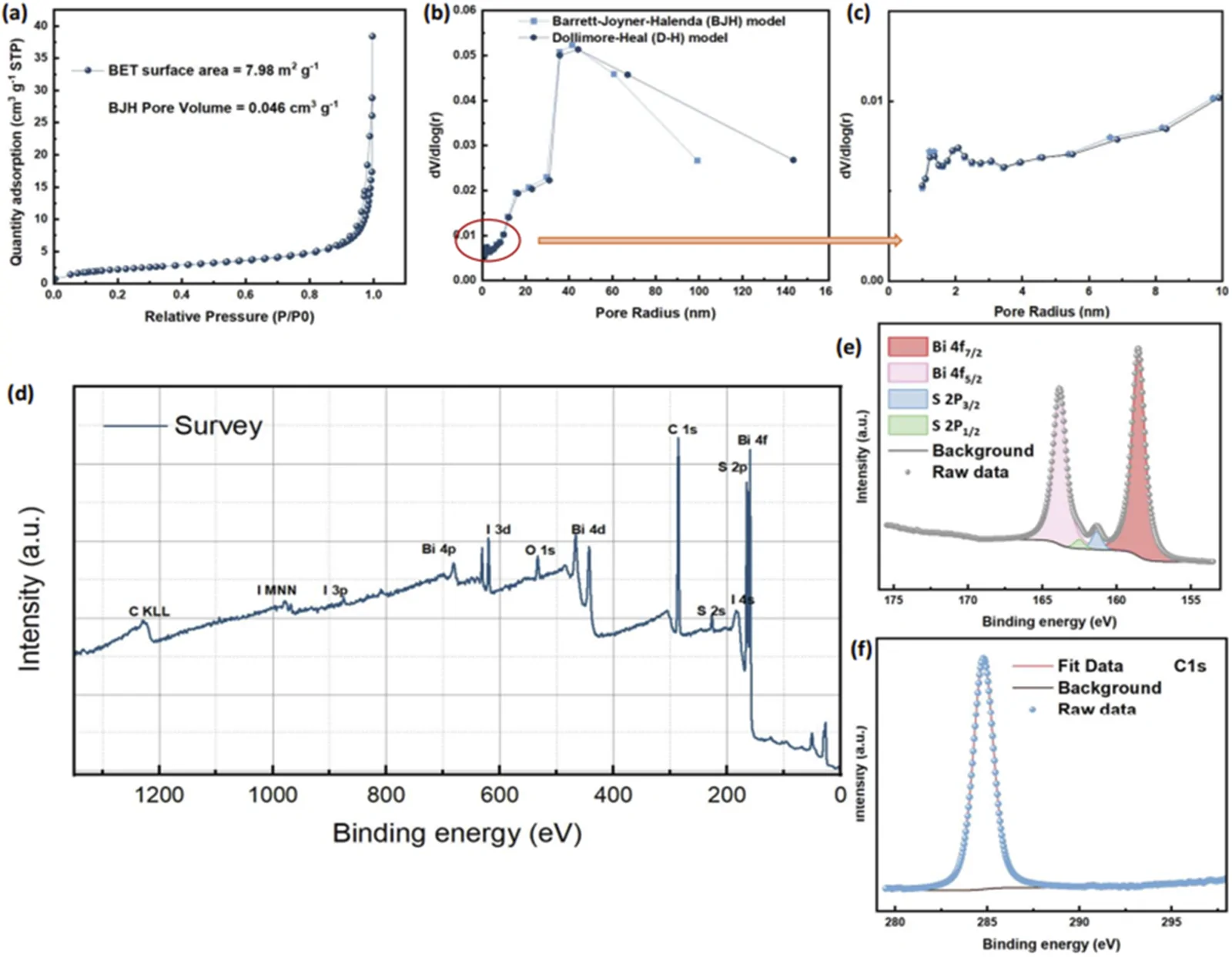
(a) N_2_ adsorption–desorption isotherms; (b) pore size distribution calculated using the BJH and D–H models; (c) enlarged view of the pore size distribution in the small pore radius range (0–10 nm); (d) X-ray photoelectron spectroscopy survey spectrum; (e) high-resolution XPS spectrum of Bi 4f and S 2p, with fitted peaks; (f) high-resolution XPS spectrum of C 1s; for the Bi_13_S_18_I_2_ sample.

The XPS survey spectrum (Fig. 2d) confirms the presence of Bi, S, and I as the primary elements. In the high-resolution Bi 4f spectrum (Fig. 2e), in addition to the Bi 4f_7/2_ and Bi 4f_5/2_ doublet, an additional feature appears in the ∼161–163 eV region, corresponding to the overlapping binding energy range of Bi 4f and S 2p signals. Owing to this overlap, the S 2p_3/2_ and S 2p_1/2_ contributions may lead to apparent peak broadening. The C 1s spectrum (Fig. 2f) exhibits a single peak at 284.8 eV, which was used for binding energy calibration.

The rate performance and cycling performance of Bi_13_S_18_I_2_ sample was studied in a half-cell configuration, with a Na metal as the counter electrode and the reference electrode. Fig. 3a compares the rate performance of Bi_13_S_18_I_2_ in different electrolytes and under different voltage windows. The 1 M NaPF_6_ in diethylene glycol dimethyl ether (diglyme) electrolyte delivers higher reversible capacities than that of 1 M NaPF_6_ in ethylene carbonate-dimethyl carbonate (EC-DMC) over the investigated current densities, particularly at high rates. The effect of the voltage window is clearly rate-dependent. While extending the window to 0–2.5 V (V *vs.* Na^+^/Na) increases the discharge capacity at low rates (∼0.1 C), the 0–2.0 V window provides more stable and accessible capacity at high rates, suggesting that the additional high voltage capacity is kinetically less accessible under fast cycling conditions. After cycling at increasing current densities and returning to 30 mA g^−1^, the diglyme-based electrolyte keeps a higher recovered capacity than that from EC-DMC, further demonstrating its better reversibility.

**Fig. 3:**
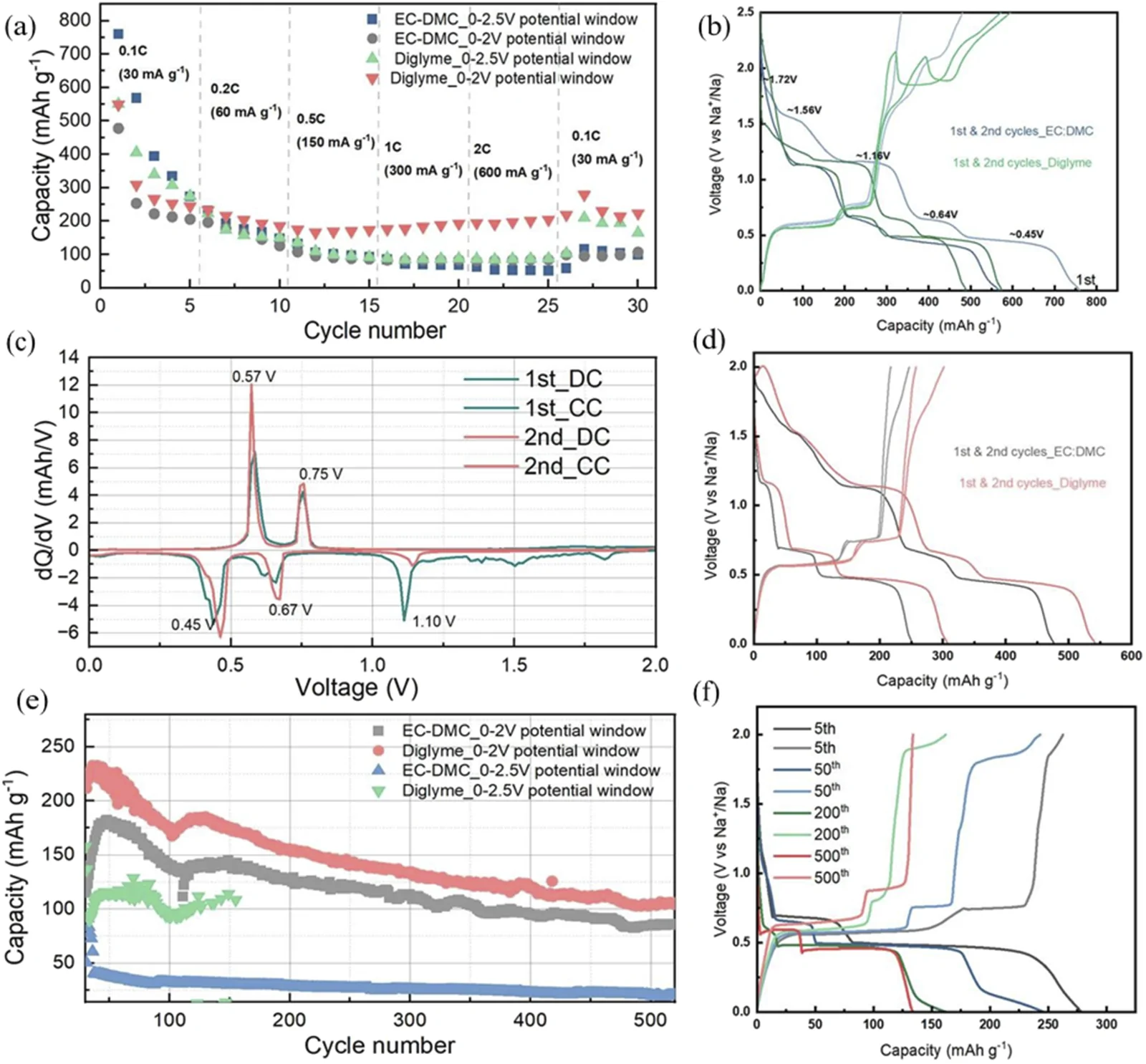
Electrochemical performance of the Bi_13_S_18_I_2_ electrode in different electrolytes and potential windows. (a) Rate performance; (b and d) voltage profiles for the first two cycles; (c) d*Q*/d*V* curves for the first and second cycles charge (CC) and discharge (DC); (e) long-term cycling performance under different electrolytes and potential windows; (f) voltage profiles at selected cycles.

The voltage–capacity profiles in Fig. 3b–d exhibit comparable multi-step features, indicating that Bi_13_S_18_I_2_ follows similar sodiation pathways in both EC-DMC and diglyme electrolytes. During discharge, characteristic plateaus can be observed at ∼1.6 V, ∼1.1 V, and in the low-potential region between ∼0.6 and ∼0.4 V. The discharge features at ∼1.6 V and ∼1.1 V are associated with other phases, which is confirmed by *ex situ* PXRD studies below (shown in Fig. 4). During the first discharge, PXRD shows the formation of metallic Bi, supporting the occurrence of conversion reactions in this voltage region. The generated Bi^0^ species subsequently participate in the alloying reaction with Na ions in the low-potential region (0.6–0.4 V), corresponding to the sequential formation of NaBi and Na_3_Bi. Upon desodiation, two plateaus are observed in the low-potential region (∼0.5–0.7 V), which are attributed to the dealloying of Na_3_Bi and NaBi back to metallic Bi, respectively. In Fig. 3d, no additional plateau is observed above 1.0 V. Consistently, the corresponding d*Q*/d*V* curves (Fig. 3c) display only two pronounced anodic peaks during desodiation, in line with the stepwise dealloying reactions. However, when the voltage window extends to 2.5 V, an additional shoulder-like feature appears between 2.0 and 2.5 V, particularly in the diglyme-based electrolyte (Fig. 3b). This feature indicates the involvement of an extra redox contribution activated under the extended voltage range.

**Fig. 4:**
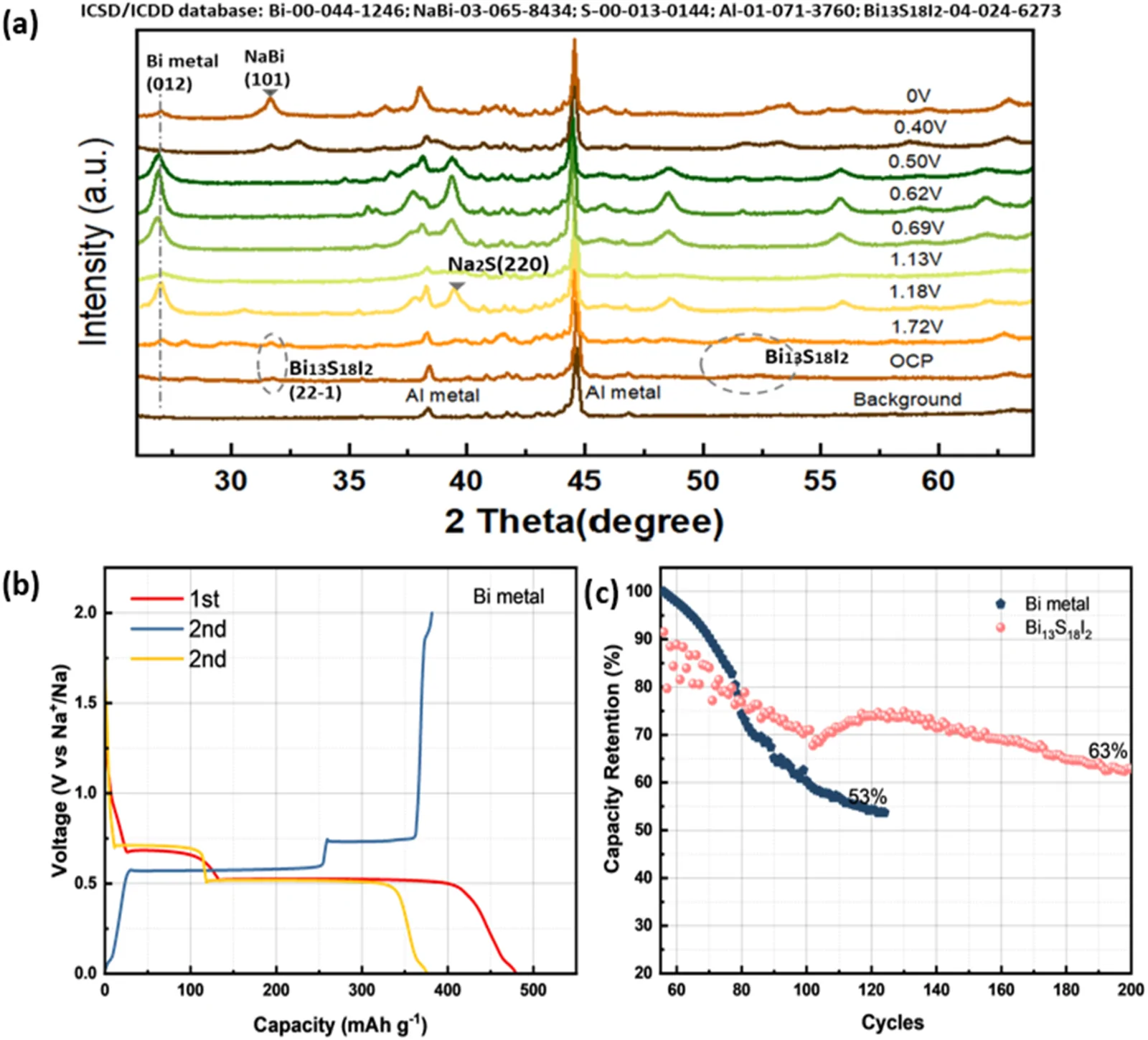
(a) *Ex situ* XRD patterns of the Bi_13_S_18_I_2_ electrode at different voltages during the first-discharge process. (b) Galvanostatic charge–discharge profiles of Bi metal electrode. (c) Cycle performance of Bi_13_S_18_I_2_ compared with Bi metal.

The long-term cycling performance in Fig. 3e further highlights the impact of the voltage window selection. The cycling stability of Bi_13_S_18_I_2_ in the 0–2.0 V window exhibits improved stability compared to those cycled between 0–2.5 V. Furthermore, the diglyme-based electrolyte within the 0–2.0 V window delivers the best durability, maintaining 133 mAh g^−1^ after 500 cycles, whereas EC-DMC under the same voltage range retains 100 mAh g^−1^. In contrast, both electrolytes lead to rapid capacity decay when the upper voltage limit is extended to 2.5 V. These results suggest that avoiding high-potential processes by limiting the window to 0–2.0 V enhances stability, particularly in the diglyme electrolyte. To further understand the electrochemical behaviours during cycling, the sodiation and desodiation profiles at selected cycles (5th, 50th, 200th and 500th) are compared in Fig. 3f. Unlike the initial sodiation process in Fig. 3d with multiple voltage features, the voltage profiles become simplified after cycling. Only two discharge plateaus and two corresponding charge plateaus are observed, which are characteristic of the reversible alloying and dealloying reactions of metallic Bi with Na.

To clarify the sodium storage mechanism, the plateau capacity contribution of Bi_13_S_18_I_2_ were quantitatively analysed and compared with those of a metallic Bi electrode in Table S4 and Fig. 4. For metallic Bi, two well-defined plateaus are observed during sodiation at ∼0.7 and ∼0.5 V, corresponding to capacities of ∼100 and ∼220 mAh g^−1^, respectively in Fig. 4b. This distribution follows the expected 1 : 2 ratio based on the stepwise Bi → NaBi → Na_3_Bi alloying mechanism. The desodiation process exhibits a nearly symmetric capacity distribution, further confirming the high and two-step electron transfer pathway.

In contrast, the Bi_13_S_18_I_2_ electrode exhibits different behaviour. The multiple voltage plateaus observed in the initial cycles indicate that the electrode is first involved in conversion reactions leading to the formation of Bi metal and Na-containing matrix. *Ex situ* XRD (Fig. 4a) confirms the formation of Na_2_S after the sodiation to 1.18 V. After the initial cycles, the electrochemical behaviour gradually stabilises into two dominant plateaus in Fig. 3f, related to Bi → NaBi → Na_3_Bi alloying reactions. Although two dominant plateaus are located at similar potentials to those of metallic Bi, their capacity distribution differs from the theoretical 1 : 2 ratio. For example, at the 5th cycle, the sodiation capacities of two plateaus are 55.4 and 154.2 mAh g^−1^, respectively. And the difference becomes more obvious at the 50th cycle (30.8 *vs.* 123.2 mAh g^−1^). A comparison of the plateau capacity ratios for both sodiation and desodiation is summarised in Table S4. The results indicate a clear difference between the sodiation and desodiation behaviours of Bi_13_S_18_I_2_.The sodiation plateau capacity distribution deviates from the ideal 1 : 2 ratio, whereas the desodiation process shows a closer resemblance to the dealloying behaviour of Bi metal. This asymmetric behaviour indicates a more complex Na-storage process in Bi_13_S_18_I_2_, although its origin cannot be conclusively determined from the present results. In addition, a weak diffraction feature assigned to NaBi remains observable at 0 V in *ex situ* XRD pattern. This may indicate incomplete conversion to Na_3_Bi, although incomplete sodiation due to kinetic limitations cannot be excluded. Together with the deviation from the ideal 1 : 2 capacity ratio, these observations suggest a more complex sodiation behaviour of Bi_13_S_18_I_2_ compared with Bi metal.

Considering the Bi content in Bi_13_S_18_I_2_, the theoretical capacity associated with complete Bi-to-Na_3_Bi alloying is approximately 295 mAh g^−1^ on a Bi_13_S_18_I_2_ mass basis. The capacity of ∼133 mAh g^−1^ after 500 cycles indicates that the theoretical alloying capacity is not fully utilized, which remains a limitation for practical applications. On the other hand, this reduced capacity utilisation may reflect the different alloying behaviour of the mixed-anion Bi_13_S_18_I_2_ compared with pure Bi. As shown in Fig. 4c, the Bi electrode undergoes rapid degradation and fails after around 120 cycles, whereas Bi_13_S_18_I_2_ maintains a higher capacity retention over 200 cycles. This comparison suggests a possible balance between capacity utilisation and cycling stability in the mixed-anion system.

## Conclusions

In summary, we demonstrate that the sodium storage behaviour of Bi-based anodes can be tuned through the introduction of a mixed-anion framework. Unlike conventional Bi metal or single-anion compounds, Bi_13_S_18_I_2_ undergoes a conversion-triggered structural reorganisation that generates the Na_2_S phase, within which subsequent alloying reactions proceed along a non-ideal pathway. More importantly, this work reveals that the local chemical environment of Bi, governed by the mixed-anion configuration, plays an important role in influencing the reaction pathway. Such a structural origin for regulating alloying behaviour provides a new perspective beyond conventional strategies that focus on nanosizing or compositing. Looking forward, this concept of coupling conversion reactions with alloying processes through tailored anion chemistry may be extended to a broader range of alloy-type electrode materials. Further exploration of mixed-anion systems with tuneable local coordination environments could open up new opportunities for designing high-performance electrodes with controlled reaction pathways and improved structural stability in SIBs and beyond.

## Author contributions

X. Y. carried out and analysed the practical work. X. Y. performed electrode preparation, electrochemical testing and *ex situ* XRD with assistance from Y. Z. and Z. G. M. G. performed BET measurements and analysis. N. R., C. K. and M. T. directly supervised this work. X. Y. wrote the paper with contributions from all authors.

## Conflicts of interest

The authors declare that they have no completing interests.

## Supplementary Material

TA-014-D6TA06219A-s001

## Data Availability

The data supporting this article have been included as part of the supplementary information (SI). Supplementary information is available. See DOI: https://doi.org/10.1039/d6ta06219a.
