# Supplementary material for: The multi-anion environment of Bi13S18I2 governs the mechanism of sodium storage
Source: J Mater Chem A Mater. 2026 Sep 21;14(62):43326–32. doi: 10.1039/d6ta06219a (PMC13621581; doi:10.1039/d6ta06219a)
Supplement: Supplementary file 1 [file TA-014-D6TA06219A-s001.pdf]

Supplementary Information (SI) for Journal of Materials Chemistry A.
This journal is © The Royal Society of Chemistry 2026

The multi-anion environment of $Bi_{13}S_{18}I_2$ governs the mechanism of sodium storage

Xuerui Yi,[a] Yuanzhu Zhao,[b] Johny (Zhenyu) Guo,[b] Megha Goyal,[c] A. Robert Armstrong,[c] John T.S. Irvine,[c] Maria-Magdalena Titirici,*[b,d] Caroline Kirk*[a] and Neil Robertson*[a]

a. School of Chemistry, EaStCHEM, University of Edinburgh, King's Buildings, David Brewster Road, Edinburgh, Scotland EH9 3FJ, UK

b. Department of Chemical Engineering, Imperial College London, South Kensington Campus, Exhibition Rd, London SW7 2AZ, UK.

c. School of Chemistry, University of St Andrews, St Andrews, Fife KY16 9ST, UK

d. Advanced Institute for Materials Research (WPI-AIMR), Tohoku University, Sendai, Japan

Experimental

Na metal (99.8% metals basis) was purchased from Alfa Aesar. Sodium carboxymethyl cellulose (CMC, average Mw 250000 powder), bismuth nitrate pentahydrate, thioacetamide, and potassium iodide were bought from Sigma-Aldrich. Sodium hexafluorophosphate (99.5%), ethylene carbonate (EC, 99.99%), dimethyl carbonate (DMC, 99.99%), diethylene glycol dimethyl ether (diglyme, 99.99%) was purchased from Guangdong Canrd New Energy Technology Ltd.

Synthesis of $Bi_{13}S_{18}I_2$ materials: 1 mmol $Bi(NO_3)_2$ $5H_2O$(0.485g) was first dispersed in 20 ml of deionized water (DI water) to form a homogeneous suspension and heated to 120 °C under continuous stirring. Separately, KI (0.03g, 0.18mmol) and thioacetamide (TAA, 0.20g, 2.7mmol) were dissolved in 20 ml of DI water and preheated to 80 °C. The $Bi(NO_3)_2$ suspension was then slowly added to the KI/TAA solution. The reaction mixture was maintained at 80 °C for 1 h to allow complete precipitation. After cooling to room temperature, the solid product was collected by centrifugation, thoroughly washed deionized water, and dried under vacuum.

Material Characterisation: Powder X-ray diffraction (PXRD) data were collected using a Rigaku MiniFlex benchtop X-ray powder diffractometer set up in reflection (Bragg-Brentano) geometry with Cu K-alpha radiation (Ni filter), 1 mm knife edge and a D/teX Ultra 0D/1D High Speed Detector, over the 2-theta range 5° to 65° using a scanning speed of 5.5° per minute. Powder samples were prepared as a thin smear on a silicon substrate. The morphologies and elemental analysis of the prepared samples were examined by Scanning Electron Microscopy (SEM) with 10kv accelerating voltage. Transmission Electron Microscopy (TEM) was performed on FEI Titan Themis operated at 200 kV and equipped with a CEOS DCOR probe corrector, a SuperX energy,and a Ceta CMOS camera. X-ray Photoelectron Spectroscopy (XPS) measurements used a monochromatized ($\Delta\varepsilon$ < 300 meV) Scienta 300 X-ray photoelectron spectrometer with X-ray source Al Kα radiation (1486.6 eV). Raman spectra were recorded on an inVia Renishaw micro-Raman spectrometer with a 514 nm laser as the excitation source from 100 to 600 cm^{-1}. The Brunauer-Emmett-Teller surface area was measured with a Micromeritics ASAP2020 analyser at -196 °C. The pore size distribution was calculated using the Barrett-Joyner-Halenda (BJH) method.

Fabrication of $Bi_{13}S_{18}I_2$ // Na coin cell: The bismuth sulfide iodine slurries were prepared by mixing 80 wt. % $Bi_{13}S_{18}I_2$, 10 wt. % super P and 10 wt. % pre-prepared sodium carboxymethyl cellulose binder solution (5 wt. %) in water. Electrode was coated from slurries onto battery-grade Cu foil with about 200 µm thickness. The coated electrodes were dried at room temperature for 4 hours followed by drying in an oven for overnight at 80 °C. The mass loading of resulting electrodes is between 1.5 mg – 2.0 mg cm^{-2}. 0.8*0.8 cm electrodes were cut and used in CR-2032-type coin cell. Regarding the coin

cell, the electrodes were assembled inside a glovebox against sodium metal disc (10mm in diameter) with a Celgard separator saturated with 1M $NaPF_6$ in diethylene glycol dimethyl ether (diglyme).

Electrochemical measurements were carried out using a Land CT2001A battery testing system and a a Biologic potentiostat. Galvanostatic charge and discharge (GCD) measurements were performed on the Land system at different current densities within the specified voltage range.

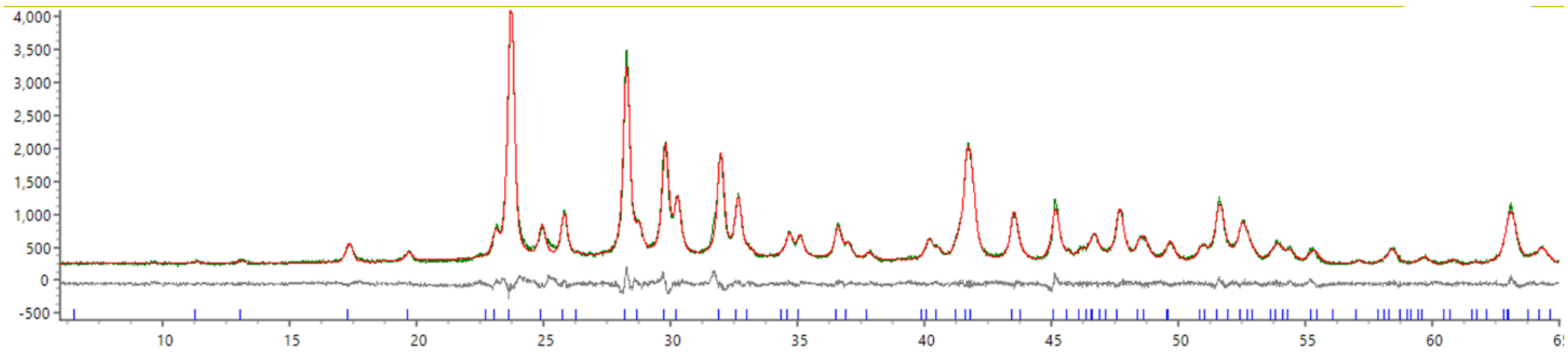

Figure S1. Pawley fit of data collected on the sample $Bi_{13}S_{18}I_2$ – green (observed), red (calculated), grey (difference) profiles. with the reference PDF card (Groom *et al* ICSD 243730).

Table S1. Comparison of Raman peak positions for $Bi_{13}S_{18}I_2$ and related Bi-based compounds.

**Raman Peak region (cm^{-1})**	**This work $Bi_{13}S_{18}I_2$**	**$Bi_{13}S_{18}I_2$**	**Bi_2S_3**	**BiI_3**
**~68-74**	✓	✓	✓ (~65)	✓
**~88**	✓	✓	-	✓ (~85)
**~106**	✓	✓	✓ (102-108)	-
**~110-180**	Weak broad	Weak broad	✓ (123,145)	✓
**~200-220**	✓	✓	-	✓
**~260-320**	✓ (~266)	✓ (~265)	✓ (246)	✓ (259-263)

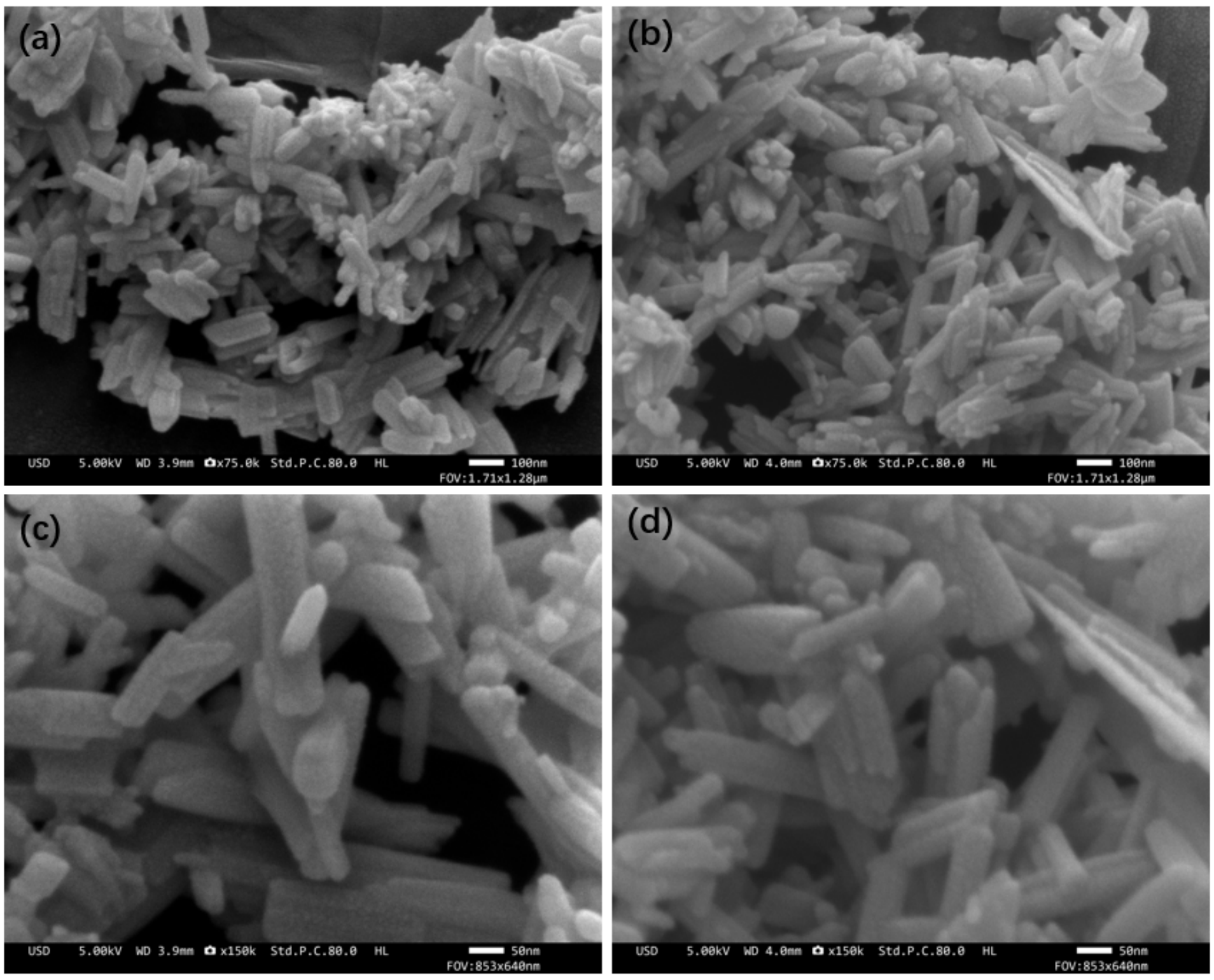

Figure S2. (a-d) High-magnification SEM images of $Bi_{13}S_{18}I_2$.

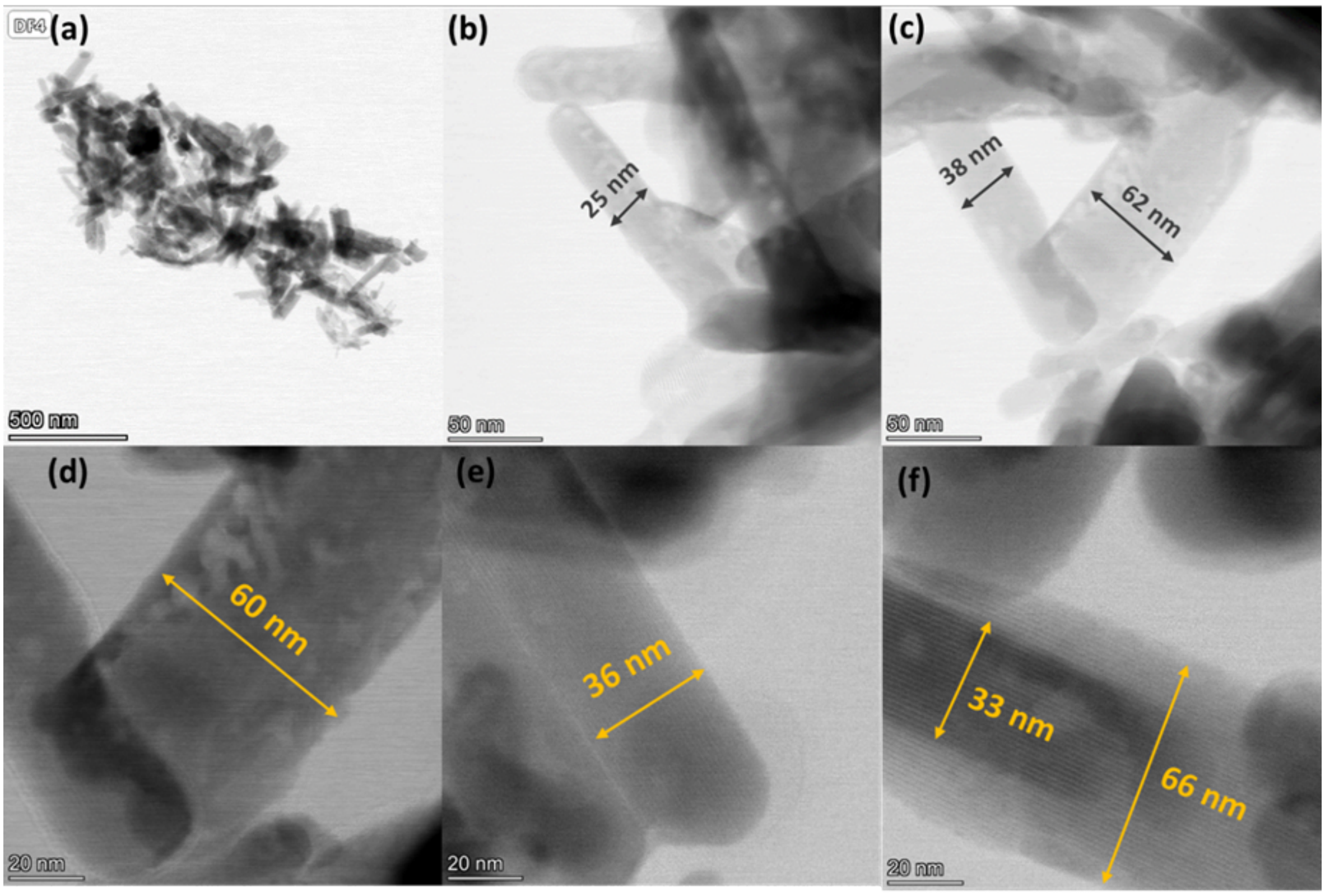

Figure S3. High-resolution TEM image of $Bi_{13}S_{18}I_2$.

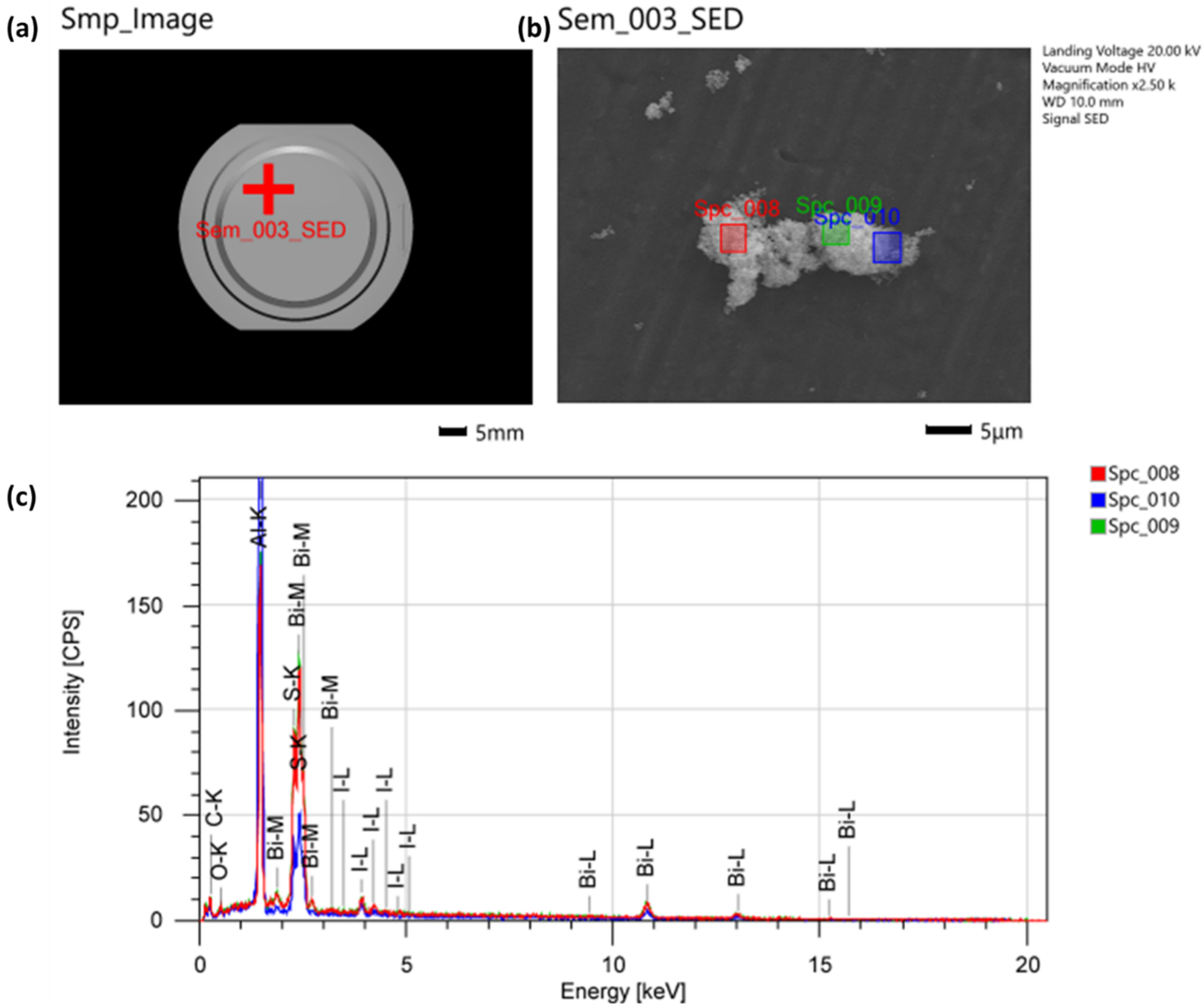

Figure S4. (a) Schematic image showing the sample position inside the SEM chamber; (b) SEM image of $Bi_{13}S_{18}I_2$ particles with selected SEM-EDS analysis spots (Spec_008, Spec_009, and Spc_010). (c) Corresponding energy-dispersive X-ray spectroscopy (EDS) spectra collected from different regions, confirming the presence of Bi, S and I element.

Table S2. Elemental composition of $Bi_{13}S_{18}I_2$ determined by SEM-EDS analysis (atom %).

Name	C	O	Al	S	I	Bi	Total
Spc_008				46.43	15.76	37.81	100.00
Spc_010				39.71	24.81	35.48	100.00
Spc_009				46.24	14.70	39.06	100.00
Average				44.13	18.42	37.45	
Standard Deviation				3.12	4.54	1.48	

Atom/Mol%

Table S3. Elemental composition of $Bi_{13}S_{18}I_2$ determined by TEM-EDS analysis (atom %).

TEM-EDS Points ($Bi_{13}S_{18}I_2$)	Elements (Atom %)			
	Bi	S	I	O
1	50.7	40.6	5.75	2.93
2	52.1	40.7	5.52	1.75
3	49.4	41.8	5.70	3.06
average	50.7	41.0	5.66	2.6
Theoretical	39.4	54.5	6.10	

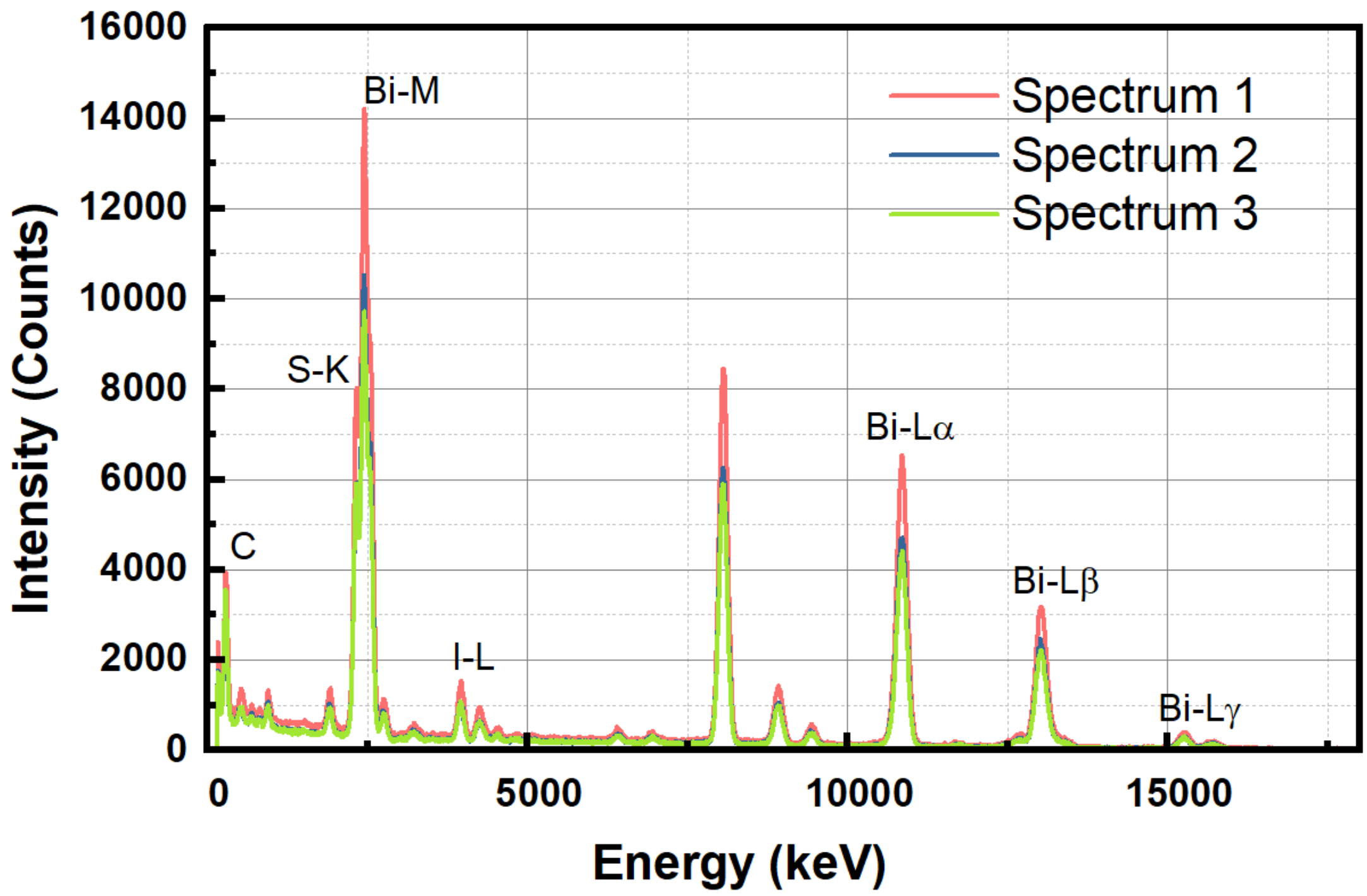

Figure S5. TEM-EDS spectra of $Bi_{13}S_{18}I_2$.

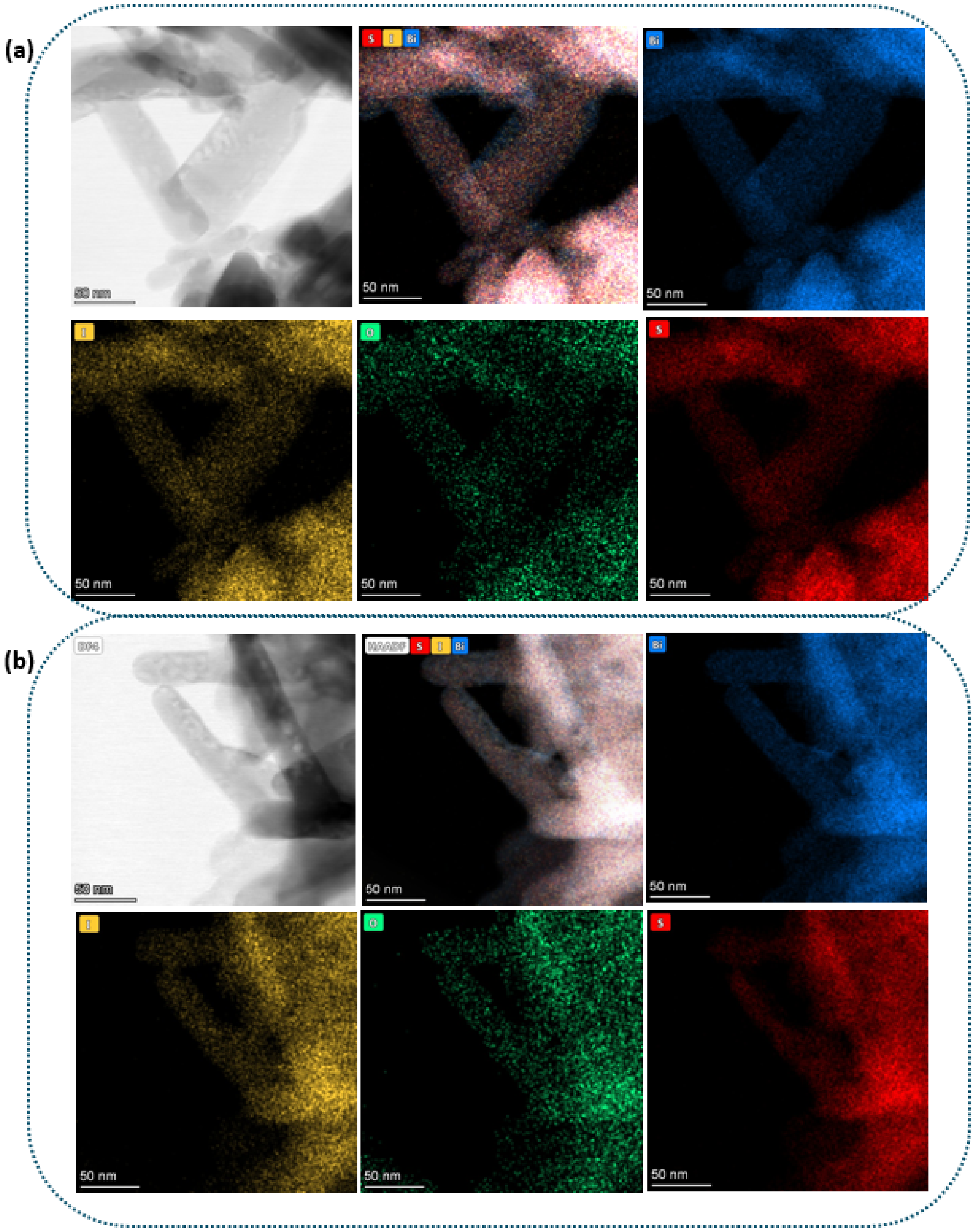

Figure S6. TEM-EDS mapping of $Bi_{13}S_{18}I_2$.

Table S4. Capacity ratios of alloying reactions during sodiation and desodiation.

Capacity (mAh g^{-1})		Bi (metal)		$Bi_{13}S_{18}I_2$ (5th)		$Bi_{13}S_{18}I_2$ (50th)		$Bi_{13}S_{18}I_2$ (200th)	
Alloying	$Bi^0 \rightarrow Bi^{1-}$	100	Ratio = 1:2.2	55	Ratio = 1:2.8	31	Ratio = 1:4.0	31	Ratio = 1:2.5
	$Bi^{1-} \rightarrow Bi^{3-}$	220		154		123		79	
Dealloying	$Bi^{3-} \rightarrow Bi^{1-}$	224	Ratio = 1:2.2	118	Ratio = 2.3	104	Ratio = 1:3.4	31	Ratio = 1:2.32
	$Bi^{1-} \rightarrow Bi^0$	101		51		30		73	
